# Cryopreservation and Thawing of *Ex Vivo* Expanded Cord Blood Hematopoietic Stem Cells

**DOI:** 10.14740/jh2167

**Published:** 2026-02-20

**Authors:** Lia Morton, Induja Arulchelvam, Chelsea McGregor, Layla Ghannouchi, Nicolas Pineault

**Affiliations:** a Canadian Blood Services, Centre for Innovation, Ottawa, ON, Canada; b Translational and Molecular Medicine, Faculty of Medicine, University of Ottawa, ON, Canada; c Biochemistry, Microbiology and Immunology Department, University of Ottawa, Ottawa, ON, Canada

**Keywords:** Cryopreservation, Thawing, Hematopoietic stem cells, Stem cell expansion, Stem cell agonist cocktail, Cell death

## Abstract

**Background:**

Umbilical cord blood (CB) is an invaluable source of hematopoietic stem and progenitor cells (HSPCs). Its use in stem cell transplantation is however constrained by the insufficient cell dose present in each unit. Recent development in *ex vivo* HSPC expansion technologies addresses this issue and encourages the use of the best matched CB unit. In this study, we sought to develop a cryopreservation and thawing protocol for *ex vivo* expanded HSPC.

**Methods:**

CB CD34^+^ HSPC-enriched cells were expanded in serum-free medium supplemented with a previously optimized mix of chromatin-modifiers and early acting cytokines for 7-days. CB HSPC were then harvested and prepared for cryopreservation. Thawed CB samples were then analyzed by flow cytometry to measure cell viability and recovery of HSPC-enriched fractions, while graft potency was measured using the colony-forming unit (CFU) assay.

**Results:**

First, we compared two widely used means of freezing; a passive isopropyl alcohol-based freezing container vs. a controlled-rate freezer (CRF). Both methods exhibited comparable recovery of viable cell numbers, including the HSC-enriched CD34^+^CD45RA^–^CD90^+^ fraction, and similar potency measured using the CFU assay. Next, we compared two thawing methods frequently used in clinical settings. The “thaw and dilute” method slightly improved the recovery of total nucleated cells (TNC) and HSPC fractions over the “rinse” method, though potency was comparable between both thaw methods. Next, we investigated the impact of three different commercial freezing solutions on product recovery. Dimethyl sulfoxide (DMSO)/dextran-40 and CryoProtectPure-STEM (CPP) provided superior recovery of HSPC-fractions and potency when compared to CryoScarless (CSL).

**Conclusions:**

Taken together, this study provides insights into alternative, less harmful options for the freezing and thawing of *ex vivo* expanded HSPCs.

## Introduction

Umbilical cord blood (CB) is a rich source of hematopoietic stem and progenitor cells (HSPCs), which can be used for stem cell transplantation [1]. A notable advantage of CB over adult stem cells is the less stringent human leukocyte antigen (HLA) matching requirements, short procurement and reduced risk of graft-vs-host disease [1, 2]. In addition, collection from minority or ethnically diverse groups helps increase the diversity of HLA inventory available [3]. However, a major drawback of CB is the low number of cells, including hematopoietic stem cells (HSCs) present per unit [3]. The reduced total nucleated cells (TNCs) and HSC dose lead to slower platelet and leukocyte engraftment, which increases the patient’s risk of treatment-related mortality [1, 4, 5].

One strategy that has provided meaningful results in clinic is the *ex vivo* expansion of CB units’ HSPC prior to transplantation. While *ex vivo* expansion of HSC was historically challenging, recent strategy that combines the use of stem cell agonists (i.e., small molecules promoting HSC self-renewal) with early acting cytokines [6] significantly raised the level of expansion of HSPC with high engraftment activity. Several of the stem cell agonists identified to date such as valproic acid, nicotinamide and UM171 act as chromatin modulating agents [4, 7, 8]. StemRegenin 1 is another stem cell agonist with a different means of action, shown to act as an aryl hydrocarbon receptor antagonist [7]. Importantly, the use of CB units expanded with nicotinamide [9], UM171 [2, 10] or StemRegenin 1 [11] was reported to accelerate engraftment in clinical trials including one phase III trial [12].

A cornerstone in the field of stem cell transplantation is the degree of match between the donor and the recipient. This is one of the most important variables to promote prompt engraftment and minimize graft-vs-host-disease [13, 14]. Such outcomes are also associated with lower treatment-related death and improved quality of life [15, 16]. An underappreciated advantage of expansion technologies is that it can allow for the use of a better-matched CB unit that would otherwise be unsuitable for transplantation due to insufficient cell dose [2, 6].

We reasoned that, as seen before with cytokines [17, 18], a combination of stem cell agonists could support superior growth and expansion of HSPCs through additive and synergistic activities. Hence, we developed a series of stem cell agonist cocktail (SCAC) through factorial and central composite design screens. The SCACs significantly improved expansion of HSPCs and provided superior engraftment [4]. The top SCAC X2A, composed of StemRegenin 1, UM171, valproic acid, and L-ascorbic acid 2-phosphate (AA2P), supported over a 2,500-fold expansion of TNC, a 1,500-fold expansion of CD34^+^ cells, and a 500-fold expansion of CD34^+^CD45RA^–^ HSPCs over a 14-day period. This strong growth was accompanied by a 15-fold expansion in the frequency of long-term SCID-repopulating cells (i.e., HSC), which was four-fold higher than that observed with the combination of StemRegenin 1 and UM171 [4]. Currently, transplantation of CB unit following *ex vivo* expansion is only done in rare cases, mostly in the context of clinical trials. The expanded HSPC fractions is transplanted directly after expansion, together with the lineage-positive fractions containing mature cells (lymphoid and myeloid). One issue with this strategy is the significant manufacturing time and the cumbersome logistic surrounding this procedure, which can delay the graft availability.

The usage of pre-expanded frozen CB grafts could be a potential solution to this problem. Besides, development of freezing protocols for expanded HSPCs would facilitate the storage and transport of the grafts to clinic sites. However, many challenges remain before such strategy is applicable, and only a few studies have investigated this topic. When Giarratana et al first investigated this option in 1998, they showed that bone marrow HSPC could be expanded in culture bags under cytokine-rich conditions, supporting the expansion of myeloid CFU and multipotent progenitor cells that could be frozen, with post-thaw recovery reported in the range of 45–90% [19]. In 2001, Rice et al reported the engraftment outcomes of cryopreserved *ex vivo* expanded CB HSPC. While the engraftment was only measured at 6 weeks post-transplant in immunodeficient mice, the study nonetheless confirmed that pre-expanded frozen grafts could support short-term engraftment as efficiently as the non-expanded grafts [20]. On the other hand, Duchez et al optimized a freezing medium for expanded CB HSPC grafts (HP01-dimethyl sulfoxide (DMSO)), which was shown to support a high engraftment level 8 weeks post-transplant [21]. Lastly, Schaniel et al recently demonstrated that clinical-grade, cryopreserved VPA-expanded CB HSPC sustained equivalent high level long-term serial engraftment in NSG mice compared with the same grafts transplanted without prior cryopreservation [22]. Together, these studies provide supporting evidence that the engraftment activity of *ex vivo* expanded HSPC is well maintained during cryopreservation.

The creation of pre-expanded, cryopreserved HSPC stocks could help decrease wait times and potentially improve transplant efficiency. Thus, the aim of the present study was to develop a procedure for the cryopreservation and thawing of pre-expanded CB HSPC. Toward this, we compared the efficiency of different freezing apparatus, of different thawing protocols and the effectiveness of three commercially available freezing solutions on the recovery of HSPC post-thaw.

## Materials and Methods

### Collection and purification of CB CD34^+^ cells

The project was preapproved by the Canadian Blood Services Ethic Review Board, and CB units were obtained following written informed consent from the Canadian Blood Services CB for Research Program. CB units contained a minimum of 750 × 10^6^ nucleated cells and were obtained less than 24 h post-collection. Mononuclear cells were isolated by Ficoll-Paque Plus (GE, Pittsburgh, PA) and CB CD34^+^ cells were enriched using the EasySep™ Human CD34 Positive Selection Kit II (StemCell Technologies, Vancouver, BC).

### Small molecules

StemReginin1 (Cat 72342), UM171 (Cat 72912) and valproic acid (Cat 72292) were purchased from StemCell Technologies and AA2P (Cat A8960) from Sigma-Aldrich (Mississauga, ON). UM171 and StemReginin1 were solubilized in DMSO while valproic acid and AA2P in phosphate-buffered saline (PBS). All molecules were aliquoted and stored at –80 °C for single use. The final concentration of DMSO in culture did not exceed 0.01% (v/v).

### Cell culture

CB CD34^+^ cells (12,000 cells/mL) were expanded for 7 days (unless indicated otherwise) in X2A cultures in a 24-well plate in serum-free medium StemSpan SFEM (Cat 09650, StemCell Technologies). The medium was supplemented with human low-density lipoprotein (Cat 02698, StemCell Technologies) at 10 µg/mL and 1% penicillin-streptomycin (Gibco), cytokines (stem cell factor, thrombopoietin and FMS-like tyrosine kinase 3 each at 100 ng/mL, PeproTech, Cranbury, NJ) and X2A (StemRegenin 1 2,500 nM, UM171 62 nM, valproic acid 0.01 mM and AA2P 1,000 µM) [4]. Cultures were maintained in humidified incubator (37 °C, 5% CO_2_) for 7 days with fresh culture medium added on day 4.

### Freezing and thawing protocol

Expanded cells were aliquoted and spun down before resuspending the pellet in 1 mL freezing media at 200,000 cells per cryovial. Pre-freeze and post-freeze viable cell counts were done manually by trypan blue staining using a hemocytometer. Unless stated otherwise, the freezing medium for all experiments was made of 5% (v/v) Human Albumin USP (Grifols Canada Therapeutics, Montreal, QC), 20% CryoSolve (55% w/v DMSO and 5% w/v dextran-40, Akron Biotechnologies), and 60% Iscove’s Modified Dulbecco’s Medium. CryoSolve was used as default freezing solution unless stated otherwise. Other freezing solutions tested included CryoProtectPure-STEM (CPP, Ad Infinitum Cell Preservation Technologies, Plymouth, MI) and CryoScarless (CSL, DiagnoCine, Hackensack, NJ). CPP was used as described above by replacing CryoSolve, while for CSL, cells were resuspended directly into CSL as indicated by manufacturer. Cryovials were frozen either in a Mr. Frosty™ freezing device (Thermo Fisher Scientific, ON, Nepean, ON) or a CryoMed controlled-rate freezer (CRF) (Thermo Fisher Scientific) following manufacturer instructions as indicated. For the CRF, the freezing program for cryovial using slow cooling consisted of the following steps: 1) ramp at 1.0 °C/min until sample = –4.0 °C; 2) ramp at 25.0 °C/min until chamber = –40.0 °C; 3) ramp at 10.0 °C/min until chamber = –12.0 °C; 4) ramp at 1.0 °C/min until chamber = –40.0 °C; 5) ramp at 10.0 °C/min until chamber = –90.0 °C. Cryovials were rapidly thawed in a 37 °C water bath then diluted 1:1 with 4% human albumin (Grifols Canada Therapeutics) in Plasma-Lyte-A (Baxter, Deerfield, IL).

### Colony-forming unit (CFU) assay

Thawed *ex vivo* expanded CB cells (1,000 cell/mL) were plated in MethoCult Classic H4434 (StemCell Technologies) and incubated at 37 °C for 14 days. Colonies were scored according to their morphology and classified as burst-forming unit-erythroid, CFU granulocyte/macrophage, and mix colonies (megakaryocyte, monocyte, granulocyte and erythroid). The total number of CFU (CFU-total) of all colony types per thawed vial is presented for each condition.

### Flow cytometry

Flow cytometry analysis was used to measure the frequency of CD34^+^ subsets and post-thaw cell viability (%) using an Attune NxT cytometer (Thermo Fisher Scientific). Unless stated otherwise, all antibodies were from Becton Dickinson Pharmingen (Mississauga, ON). The antibodies used included CD34-phycoerythrin (PE, clone clone 581), CD45RA-allophycocyanin (APC, clone H100) and CD90-PECy7 (clone 5E10). Cells were stained with antibodies and Sytox AADvanced (Thermo Fisher Scientific) for 20 min and diluted in fluorescence-activated cell sorting (FACS) buffer (PBS + 2% fetal bovine serum (FBS)). Antibodies were titrated before use, while fluorescent-minus-one controls were used to set gates and/or quadrants. The following CD34^+^ subsets were quantified by FACS: HSC-enriched (CD34^+^CD45RA^–^CD90^+^), HSPC (CD34^+^CD45RA^–^), and CD34^+^ cells.

### Statistical analysis

Data are shown as mean values ± standard error of the mean (SEM) of four independent experiments done from three different donors. Prism 9 (GraphPad, La Jolla, CA) was used to perform statistical analyses. Two-group comparisons were performed using paired two-tailed *t*-tests. Multigroup comparisons were performed using repeated measures one-way analysis of variance (ANOVA) or two-way ANOVA as indicated. A P value of less than 0.05 was considered as statistically significant.

## Results

### Experimental design overview

**Figure 1:**
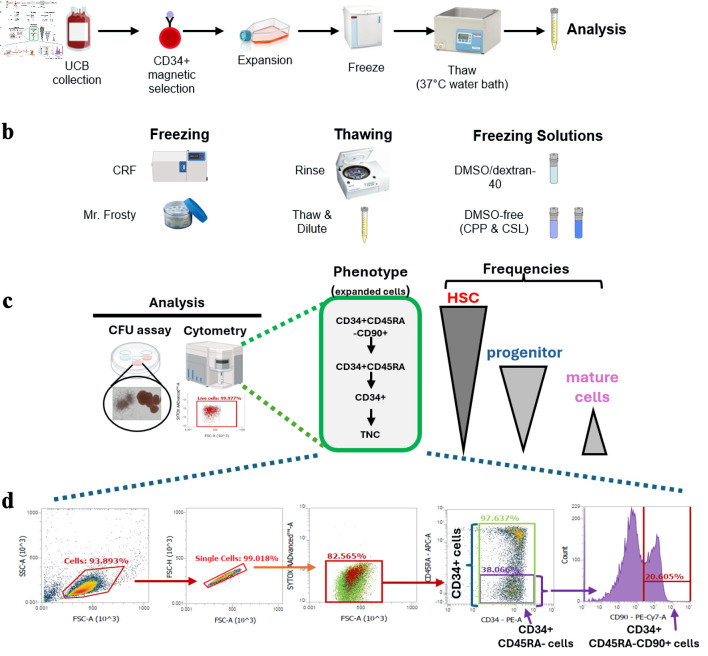
Overview of the experimental design and HSPC-fractions investigated. (a) Overview of experimental design. (b) Cryovariables investigated in this work. (c) Summary of analyses done on samples post-thaw. HSPC-fractions tracked herein by flow cytometry and their relative content in HSC, progenitors and mature cells, are presented. (d) Representative flow cytometry analyses of *ex vivo* expanded CD34^+^ cells post-expansion before freezing. Gating strategy used to track viable cells and frequency of CD34^+^-subfractions are presented. HSPC: hematopoietic stem and progenitor cell; UCB: umbilical cord blood; CRF: controlled-rate freezer; CPP: CryoProtectPure-STEM; CSL: CryoScarLess; CFU: colony-forming unit; TNC: total nucleated cell; HSC: hematopoietic stem cell; DMSO: dimethyl sulfoxide.

We set out to test various variables and approaches for the optimization of a freeze and thaw protocol for *ex vivo* expanded HSPC. CB CD34^+^ HSPC-enriched cells were expanded in X2A-supplemented, serum-free medium cultures for 7 days, after which cells were harvested, frozen, and later thawed for analyses (Fig. 1a). Different cryo-related variables were tested (Fig. 1b), which included freezing devices, thawing procedures and the use of different cryosolutions. The impact of these variables on the viability and recovery of HSPC subpopulations were measured by flow cytometry, and functional assessment of HSPC activity was assessed using the CFU assay, respectively (Fig. 1c). Representative flow cytometry analyses of CD34^+^, CD34^+^CD45RA^–^ and CD34^+^CD45RA^–^CD90^+^ cell subpopulations progressively enriched in HSC activity post-expansion are presented in Figure 1d.

### Slow cooling rate provides reliable cryopreservation outcomes for pre-expanded HSPCs

Slow cooling rates during freezing are recommended for many mammalian cells, including hematopoietic cells. Herein, we compared two different means of freezing: a CRF vs. an alcohol-based freezing device (Mr. Frosty™).

**Figure 2:**
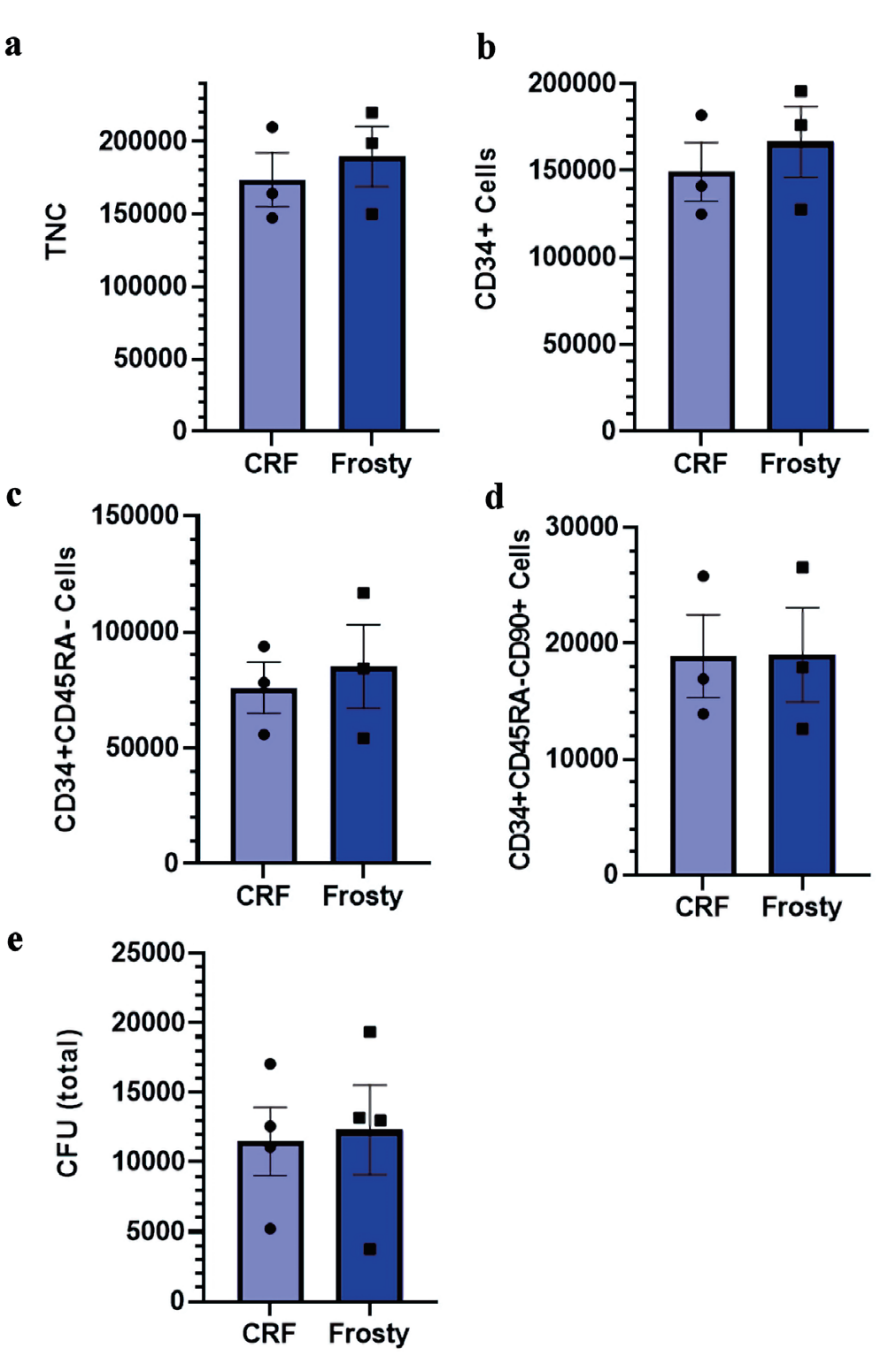
Impact of different freezing procedures on the viability and recovery of *ex vivo* expanded cryopreserved HSPCs. (a–d) Total number of nucleated cells, CD34^+^ cells, CD34^+^CD45RA^–^ cells, and CD34^+^CD45RA^–^CD90^+^ cells post-thaw in indicated conditions. (e) Total number of CFU post-thaw. Data are presented as mean ± standard error of the mean (SEM, n = 3–4). No significant differences are detected. HSPC: hematopoietic stem and progenitor cell; CRF: controlled-rate freezer; CFU: colony-forming unit; TNC: total nucleated cell.

The viabilities of TNC (mean of 93% (90–96%) and 93% (90–98%) for the CRF and Mr. Frosty, respectively), and all CD34^+^ cell fractions post-thaw (e.g., mean of 94% (91–97%) and 95% (93–99%) for CD34^+^ cells) measured by cytometry were not significantly different between the two different freezing devices (data not shown). The recovery of viable cells post-thaw, presented as total number of viable TNC, CD34^+^, CD34^+^CD45RA^–^ and CD34^+^CD45RA^–^CD90^+^ (most enriched in HSC), was also similar whether the expanded HSPCs were frozen in a CRF or a Mr. Frosty device (Fig. 2a–d). Functional assessment of HSPC post-thaw measured with the CFU assay confirmed that both apparatuses supported similar potency post-thaw (Fig. 2e). In short, these results confirm that both freezing devices provide adequate cryopreservation efficiencies for *ex vivo* expanded HSPCs. The remainder of the work presented herein was carried out using the Mr. Frosty device.

### Impact of thawing methods on viability and HSPC recovery

The most common method employed when thawing umbilical CB grafts is to simply dilute the cells to reduce DMSO cytotoxicity and avoid cell loss [23]. An alternative method is to wash the cells post-thaw through centrifugation and resuspension of the cell pellet. While this method ensures the complete removal of DMSO, it is susceptible to cell loss. We therefore chose to compare these two methods to assess their impact on the recovery of *ex vivo* expanded CD34^+^ cells post-thaw.

**Figure 3:**
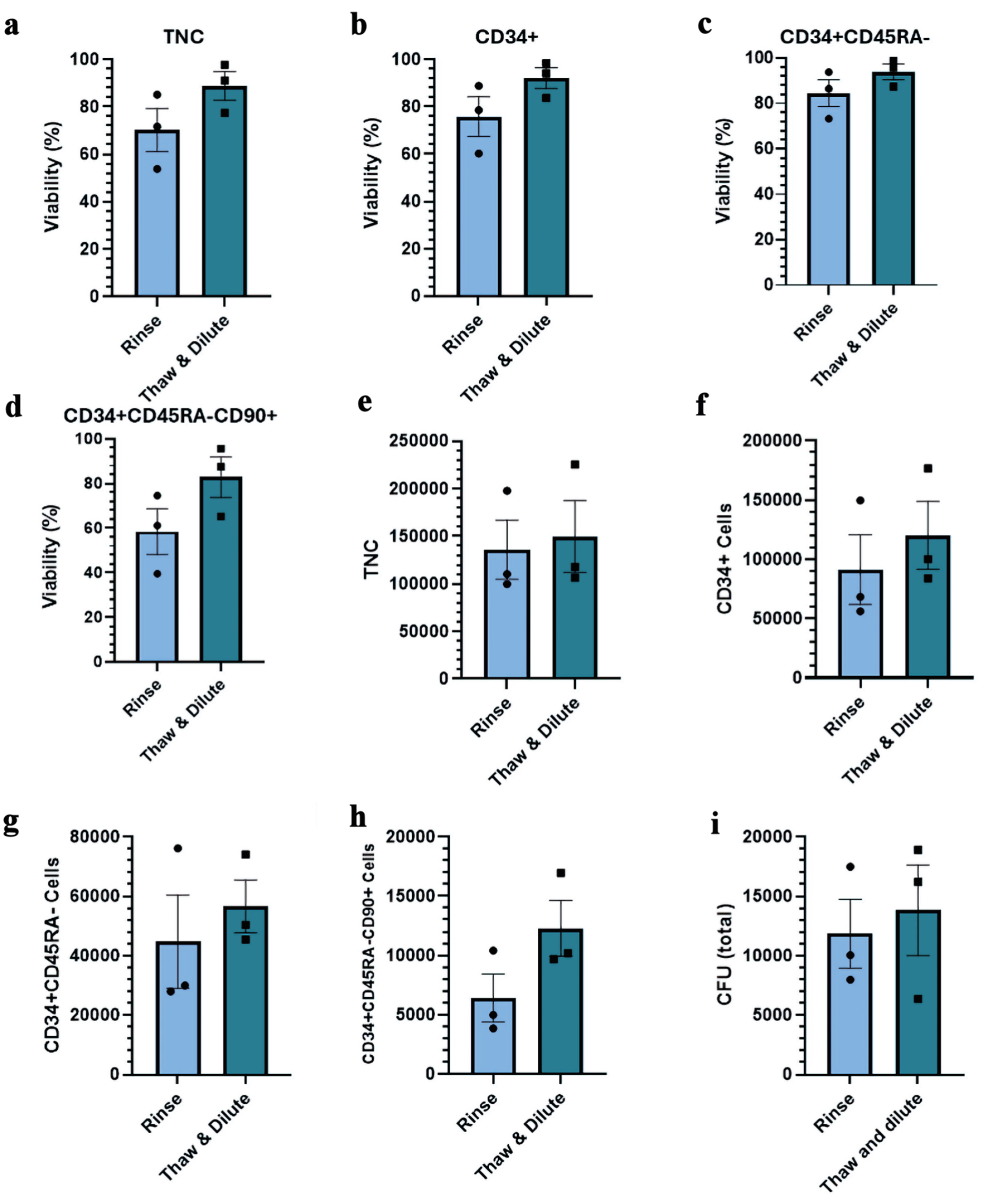
Impact of different thawing methods on the viability and recovery of *ex vivo* expanded cryopreserved HSPCs. (a–d) Viability of TNC, CD34^+^ cells, CD34^+^CD45RA^–^ cells, and CD34^+^CD45RA^–^CD90^+^ cells post-thaw. (e–h) Total number of indicated viable cells post-thaw. (i) Total number of CFU post-thaw. Data are presented as mean ± standard error of the mean (SEM, n = 4). No significant differences are detected. HSPC: hematopoietic stem and progenitor cell; CFU: colony-forming unit; TNC: total nucleated cell.

Samples thawed using the “rinse” method had a tendency of lower cell viability post-thaw though none of the differences reached statistical significance (Fig. 3a–d). As expected, we observed lower recovery of TNCs and CD34^+^ cell fractions post-thaw, including the CFU content, though the differences remain below statistical significance (Fig. 3e–i). This is consistent with the known risk of cell loss during a wash step [23]. Given the inherent risk of the “rinse” method, the remainder of this work was done with the “thaw and dilute” method.

### Impact of different freezing solutions

The next variable that we sought to test was the freezing solution. The current standard cryoprotectant for HSC grafts is DMSO [24]. However, DMSO-free cryosolutions are of interest given DMSO’s side effects that include nausea, vomiting, and abdominal cramps, as well as potential negative cardiovascular and respiratory side effects [25, 26]. Recently, we demonstrated successful cell recovery and function post-thaw using the DMSO-free cryosolutions CSL and CPP [24]. We thus decided to evaluate the impact of these two DMSO-free cryosolutions on *ex vivo* expanded CD34^+^ cells post-thaw.

**Figure 4:**
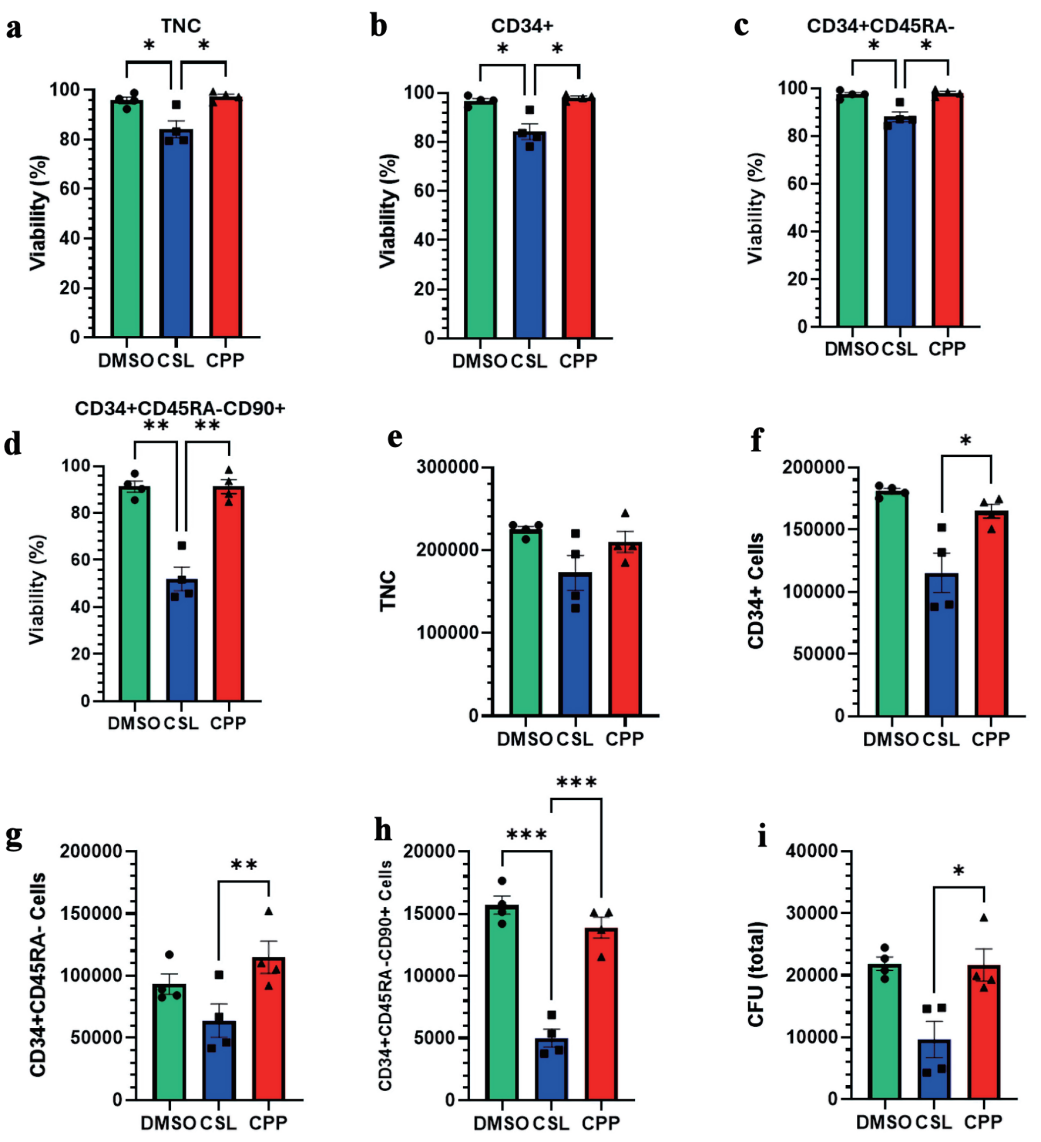
Impact of different freezing solutions on the viability and recovery of *ex vivo* expanded cryopreserved HSPCs. (a–d) Viability of TNC, CD34^+^ cells, CD34^+^CD45RA^–^ cells, and CD34^+^CD45RA^–^CD90^+^ cells post-thaw. (e–h) Total number of indicated viable cells post-thaw. (i) Total number of CFU post-thaw. Data are presented as mean ± standard error of the mean (SEM, n = 3–4). Significant differences were determined by one-way ANOVA; *P < 0.05, **P < 0.01 and *** P < 0.001. HSPC: hematopoietic stem and progenitor cell; CPP: CryoProtectPure-STEM; CSL: CryoScarLess; CFU: colony-forming unit; TNC: total nucleated cell; DMSO: dimethyl sulfoxide; ANOVA: analysis of variance.

For this, we compared CSL and CPP to the DMSO-containing solution CryoSolve, which was used up to now in this work. The cell viabilities obtained with samples cryopreserved with CryoSolve and CPP were significantly superior to those obtained with CSL. This was observed for all cell fractions investigated, including the HSC-enriched CD34^+^CD45RA^–^CD90^+^ cells (Fig. 4a–d). In line with these results, we observed lower numbers of TNCs and CD34^+^ cells post-thaw in CSL samples compared to CPP and CryoSolve (Fig. 4e, f). Moreover, CSL exhibited significantly lower numbers of CD34^+^CD45RA^–^ HSPC, CD34^+^CD45RA^–^CD90^+^ HSC and CFU post-thaw than other samples (Fig. 4g–i). Collectively, the data suggest that with *ex vivo* expanded HSPC, CPP and CryoSolve represent the better options for freezing solutions.

### Investigation of freezing solutions on delayed-onset cell death of CD34^+^ cells

**Figure 5:**
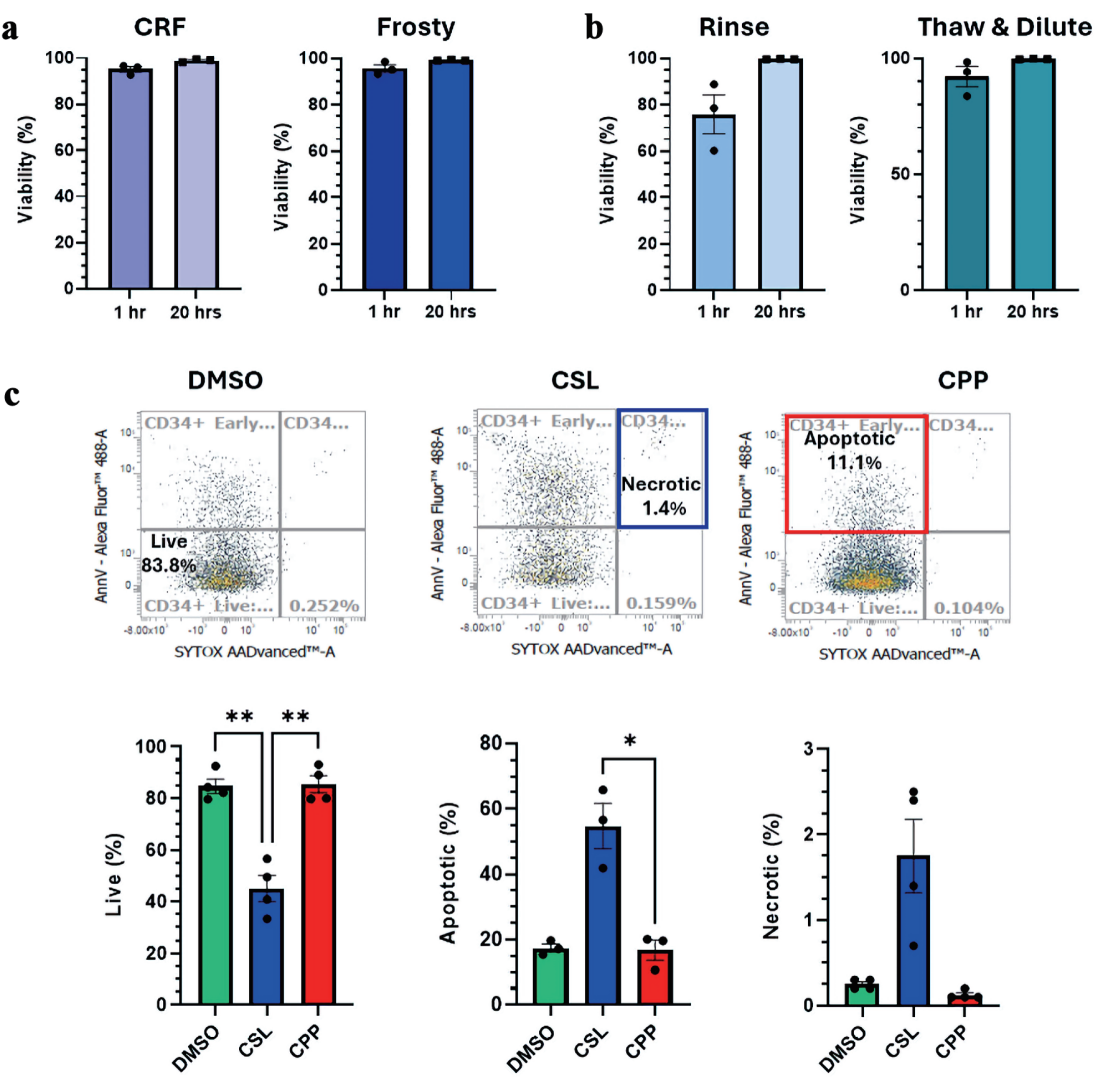
Investigation of delayed-onset cell death. (a, b) Viability measured by flow cytometry by Sytox exclusion 1.5 h and 20 h post-thaw. Comparisons between the different freezing procedures (a) and different thawing procedures (b) are presented. (c) Impact of different cryosolutions on the incidence of necrosis and apoptosis 20 h post-thaw. Representative cytometry analyses for each cryosolutions tested and summarized proportions of live, apoptotic and necrotic cells are presented. Data are presented as mean ± standard error of the mean (SEM, n = 4). Significant differences were determined by one-way ANOVA; *P < 0.05, **P < 0.01 and ***P < 0.001. CRF: controlled-rate freezer; CPP: CryoProtectPure-STEM; CSL: CryoScarLess; DMSO: dimethyl sulfoxide; ANOVA: analysis of variance.

Previous results presented viabilities and cell recoveries approximatively 1.5-h post-thaw, which does not take into consideration potential cell loss due to delayed-onset cell death. This stress response to cryopreservation and thawing can manifest itself hours or even days after thawing [27, 28]. To investigate this, samples used in previous comparisons were placed back into culture, and their viability was reassessed 20 h post-thaw. As shown, no significant decrease in the viability of the CD34^+^ cell subset was observed when the latter was assessed by DNA stain exclusion criterium (Fig. 5a, b). This is not surprising, considering that our study was done with HSPCs that contained very few mature cells, which are known to be much more sensitive to delayed-onset cell death [29–31].

Next, we assessed whether samples frozen with the different cryosolutions would show different viability profiles 20 h post-thaw when investigated by AnnexinV/Sytox staining. Viability profiles of CPP and CryoSolve samples were remarkably similar and diverged significantly from CSL samples. The profiles are consistent with what was observed post-thaw, with live CD34^+^ cells (Sytox-AnnexinV-) significantly superior in CryoSolve or CPP (Fig. 5c). Loss of cell viability in all samples was mostly attributable to apoptosis followed by necrosis, with both being superior in CSL samples (Fig. 5c).

## Discussion

*Ex vivo* expansion procedures are now making possible the use of smaller better matched units that would otherwise be unsuitable for transplantation due to insufficient cell dose. Expanded grafts can be shipped either fresh or frozen depending on product specification. An extension of this technology would be to bank pre-expanded CB HSPC, especially for units from underrepresented racial and ethnic minorities, which face the biggest challenge in finding a suitable donor due to their more diverse HLA polymorphisms. Several related proof-of-concept studies were recently reported, supporting further development [20–22]. Herein, we sought to complement these studies by focusing on the cryopreservation and thawing aspects.

Historical studies done with primary grafts paved the way for this work by optimizing several aspects for the cryogenic storage of hematopoietic grafts, such as the rate of freezing, cryoprotectants, thawing methods and many more [23, 24, 32–35]. The first step investigated in this study for the cryopreservation of expanded HSPCs was the selection of the freezing method, by comparing two frequently used apparatuses, CRF vs. Mr. Frosty. Each of these comes with their own advantages and inconveniences. CRFs are frequently used in clinical settings as they ensure reliable, reproducible and traceable freezing procedures. However, due to their cost of procurement and usage, not all institutions have access to such equipment. On the other hand, devices such as Mr. Frosty are much cheaper and can be used to freeze samples at a constant slow freeze rate. Mr. Frosty is also very easy to use, but the rate of cooling cannot be modified and is dependent on users following instructions and on the device being well maintained (e.g., change of the isopropyl solution). Samples frozen in the CRF or Mr. Frosty device showed no significant differences in the recovery of viable TNC, CD34^+^ fractions enriched in HSPC, or CFU content post-thaw. These results with expanded HSPCs are consistent with previous studies and reflect the widespread popularity of both devices [34, 36].

Next, we compared the impact of two different thawing methods on post-thaw outcomes: the “rinse” vs. the “thaw and dilute” methods. The main advantage of the “rinse” method is that it protects the graft and the patients from the harmful and side effects associated with exposure to high concentration of DMSO. A popular alternative is to dilute samples sufficiently to prevent the osmotic cytotoxic effects of DMSO on the graft and reduce infusion-related side effects [23, 34]. In this work, the “rinse” method consistently exhibited a trend of lower cell viability and cell numbers compared to the “thaw and dilute” method. This is consistent with previous work showing that the centrifugation and resuspension step can contribute to cell loss [23, 34]. Of note, in this work, samples were never exposed to high concentration of DMSO for long periods, as sample preparation for cell viability analysis started about 0.5 h post-thaw and led to sample dilution 20 mins later. Moreover, overnight incubation for the measurement of delayed-onset cell death was done after the complete removal of cryoprotectants.

Lastly, we set out to compare the efficiencies of three different freezing solutions for the cryoprotection of expanded HSPCs. In this, we compared CryoSolve (DMSO/dextran-40) to two DMSO-free solutions, CSL and CPP. CryoSolve and CPP were found equivalent and provided the highest recoveries of HSPC subsets and potency post-thaw, significantly superior to CSL. Furthermore, CSL samples were associated with a much lower percentage of live CD34^+^ cells 20 h post-thaw, due to very high levels of apoptosis and, to a lesser extent, necrosis. The significant reductions in the recovery of the CD34^+^CD45RA^–^CD90^+^ subset enriched in HSC and CFU, insinuate that the engraftment activity of CSL-frozen expanded HSPC grafts would very likely be reduced [37].

The reduced viabilities and yields observed with CSL were unexpected, considering our previous investigations in which CSL had high protective activities [24, 29]. However, our recent study discovered that the capacity of the same freezing solutions to protect HSPCs from transient warming events diverged significantly, with CSL and CPP providing the best and lowest protection, respectively [29]. Altogether, this reinforces the importance of carefully screening cryosolutions when freezing samples that have undergone significant modification. The different outcomes between the current study and the previous ones could be attributed to several factors. For one, the first two studies were done with primary CB leucocyte-enriched samples whereas the current one was done with expanded HSPCs. Cells acquire different physical properties during culture such as increased cell size, changes in membrane lipid composition and alteration of membrane permeability to name a few [38, 39]. These factors can alter the sensitivity of cells to osmotic shock, ice formation and dehydration. In second, it could be due in part to the fact that CSL is a non-concentrated solution, and samples must be resuspended directly in CSL. As such the freezing medium composition between the CPP/CryoSolve (IMDM/human albumin) and CSL (protein and serum-free formulations with carboxylated poly-L-lysine) differed significantly.

The findings of this study must be interpreted in light of several limitations. One is the limited sample size, which likely contributed to some of the differences failing to reach statistical significance. In addition, only one culture condition was used in this work. It would be interesting in future work to explore and compare the impact of different stem cell agonists or SCAC, and of different expansion times on the quality and potency of CB grafts post-thaw. Also, our current study is still in pre-clinical stage, future translation work will be required to confirm results with whole CB unit and clinical-grade consumables. Lastly, xenotransplantation will be required to confirm long-term repopulating activity of the hematopoietic grafts post-thaw ahead of future clinical translation. However, the detection of CFU in all samples observed herein provides reassurance that functional HSPCs are likely retained [37].

In summary, the present study provide support for the use of two popular freezing devices (CRF or Mr. Frosty), the use of the “thaw and dilute” method, and CPP or DMSO for freezing solutions for the cryopreservation of *ex vivo* expanded CB HSPCs. This work complements previous studies, potentially leading to the development of a cryopreserved, *ex vivo* expanded HSPC products expressing rare HLA, thereby increasing the availability of stem cell products for patients.

## Data Availability

The authors declare that data supporting the findings of this study are available within the article.

## Abbreviations

CB: cord blood
CFU: colony-forming unit
CRF: controlled-rate freezer
CPP: CryoProtectPure-STEM
DMSO: dimethyl sulfoxide
CSL: CryoScarless
HSC: hematopoietic stem cell
HSPC: hematopoietic stem and progenitor cell
HLA: human leukocyte antigen
SCAC: stem cell agonist cocktail

## References

[1] Zhu X, Tang B, Sun Z (2021). Umbilical cord blood transplantation: Still growing and improving. Stem Cells Transl Med.

[2] Cohen S, Bambace N, Ahmad I, Roy J, Tang X, Zhang MJ, Burns L (2023). Improved outcomes of UM171-expanded cord blood transplantation compared with other graft sources: real-world evidence. Blood Adv.

[3] Milano F, Thur LA, Blake J, Delaney C (2022). Infusion of non-HLA-Matched Off-the-Shelf ex vivo expanded cord blood progenitors in patients undergoing cord blood transplantation: result of a phase II clinical trial. Front Cell Dev Biol.

[4] Manesia JK, Maganti HB, Almoflehi S, Jahan S, Hasan T, Pasha R, McGregor C (2023). AA2P-mediated DNA demethylation synergizes with stem cell agonists to promote expansion of hematopoietic stem cells. Cell Rep Methods.

[5] Cheung AM, Leung D, Rostamirad S, Dhillon K, Miller PH, Droumeva R, Brinkman RR (2012). Distinct but phenotypically heterogeneous human cell populations produce rapid recovery of platelets and neutrophils after transplantation. Blood.

[6] Pineault N, Abu-Khader A (2015). Advances in umbilical cord blood stem cell expansion and clinical translation. Exp Hematol.

[7] Boitano AE, Wang J, Romeo R, Bouchez LC, Parker AE, Sutton SE, Walker JR (2010). Aryl hydrocarbon receptor antagonists promote the expansion of human hematopoietic stem cells. Science.

[8] Fares I, Chagraoui J, Gareau Y, Gingras S, Ruel R, Mayotte N, Csaszar E (2014). Cord blood expansion. Pyrimidoindole derivatives are agonists of human hematopoietic stem cell self-renewal. Science.

[9] Horwitz ME, Chao NJ, Rizzieri DA, Long GD, Sullivan KM, Gasparetto C, Chute JP (2014). Umbilical cord blood expansion with nicotinamide provides long-term multilineage engraftment. J Clin Invest.

[10] Cohen S, Roy J, Lachance S, Delisle JS, Marinier A, Busque L, Roy DC (2020). Hematopoietic stem cell transplantation using single UM171-expanded cord blood: a single-arm, phase 1-2 safety and feasibility study. Lancet Haematol.

[11] Wagner JE, Brunstein CG, Boitano AE, DeFor TE, McKenna D, Sumstad D, Blazar BR (2016). Phase I/II trial of StemRegenin-1 expanded umbilical cord blood hematopoietic stem cells supports testing as a stand-alone graft. Cell Stem Cell.

[12] Horwitz ME, Stiff PJ, Cutler C, Brunstein C, Hanna R, Maziarz RT, Rezvani AR (2021). Omidubicel vs standard myeloablative umbilical cord blood transplantation: results of a phase 3 randomized study. Blood.

[13] Loiseau P, Busson M, Balere ML, Dormoy A, Bignon JD, Gagne K, Gebuhrer L (2007). HLA Association with hematopoietic stem cell transplantation outcome: the number of mismatches at HLA-A, -B, -C, -DRB1, or -DQB1 is strongly associated with overall survival. Biol Blood Marrow Transplant.

[14] Lee SJ, Klein J, Haagenson M, Baxter-Lowe LA, Confer DL, Eapen M, Fernandez-Vina M (2007). High-resolution donor-recipient HLA matching contributes to the success of unrelated donor marrow transplantation. Blood.

[15] Keating AK, Langenhorst J, Wagner JE, Page KM, Veys P, Wynn RF, Stefanski H (2019). The influence of stem cell source on transplant outcomes for pediatric patients with acute myeloid leukemia. Blood Adv.

[16] Sharma P, Purev E, Haverkos B, Pollyea DA, Cherry E, Kamdar M, Mark T (2020). Adult cord blood transplant results in comparable overall survival and improved GRFS vs matched related transplant. Blood Adv.

[17] Audet J, Miller CL, Eaves CJ, Piret JM (2002). Common and distinct features of cytokine effects on hematopoietic stem and progenitor cells revealed by dose-response surface analysis. Biotechnol Bioeng.

[18] Pineault N, Cortin V, Boyer L, Garnier A, Robert A, Therien C, Roy DC (2011). Individual and synergistic cytokine effects controlling the expansion of cord blood CD34(+) cells and megakaryocyte progenitors in culture. Cytotherapy.

[19] Giarratana MC, Kobari L, Neildez Nguyen TM, Firat H, Bouchet S, Lopez M, Gorin NC (1998). Cell culture bags allow a large extent of ex vivo expansion of LTC-IC and functional mature cells which can subsequently be frozen: interest for a large-scale clinical applications. Bone Marrow Transplant.

[20] Rice AM, Wood JA, Milross CG, Collins CJ, Case J, Nordon RE, Vowels MR (2001). Prior cryopreservation of ex vivo-expanded cord blood cells is not detrimental to engraftment as measured in the NOD-SCID mouse model. J Hematother Stem Cell Res.

[21] Duchez P, Chevaleyre J, Brunet de la Grange P, Vlaski M, Boiron JM, Wouters G, Ivanovic Z (2013). Cryopreservation of hematopoietic stem and progenitor cells amplified ex vivo from cord blood CD34+ cells. Transfusion.

[22] Schaniel C, Papa L, Meseck ML, Kintali M, Djedaini M, Zangui M, Iancu-Rubin C (2021). Evaluation of a clinical-grade, cryopreserved, ex vivo-expanded stem cell product from cryopreserved primary umbilical cord blood demonstrates multilineage hematopoietic engraftment in mouse xenografts. Cytotherapy.

[23] Regan DM, Wofford JD, Wall DA (2010). Comparison of cord blood thawing methods on cell recovery, potency, and infusion. Transfusion.

[24] Kaushal R, Jahan S, McGregor C, Pineault N (2022). Dimethyl sulfoxide-free cryopreservation solutions for hematopoietic stem cell grafts. Cytotherapy.

[25] Stroncek DF, Fautsch SK, Lasky LC, Hurd DD, Ramsay NK, McCullough J (1991). Adverse reactions in patients transfused with cryopreserved marrow. Transfusion.

[26] Berz D, McCormack EM, Winer ES, Colvin GA, Quesenberry PJ (2007). Cryopreservation of hematopoietic stem cells. Am J Hematol.

[27] Baust JM, Snyder KK, Van Buskirk RG, Baust JG (2022). Assessment of the impact of post-thaw stress pathway modulation on cell recovery following cryopreservation in a hematopoietic progenitor cell model. Cells.

[28] Bissoyi A, Nayak B, Pramanik K, Sarangi SK (2014). Targeting cryopreservation-induced cell death: a review. Biopreserv Biobank.

[29] McGregor C, Saleem AM, Ghannouchi L, Morton L, Li M, Ben RN, Pineault N (2026). Very cold but still too warm; functional characterization of transient warming events in stem cell grafts. Cytotherapy.

[30] Xiao M, Dooley DC (2003). Assessment of cell viability and apoptosis in human umbilical cord blood following storage. J Hematother Stem Cell Res.

[31] Wang LS, Liu HJ, Xia ZB, Broxmeyer HE, Lu L (2000). Expression and activation of caspase-3/CPP32 in CD34(+) cord blood cells is linked to apoptosis after growth factor withdrawal. Exp Hematol.

[32] Perez-Oteyza J, Bornstein R, Corral M, Hermosa V, Alegre A, Torrabadella M, Ramos P (1998). Controlled-rate versus uncontrolled-rate cryopreservation of peripheral blood progenitor cells: a prospective multicenter study. Group for Cryobiology and Biology of Bone Marrow Transplantation (CBTMO), Spain. Haematologica.

[33] Kawano Y, Lee CL, Watanabe T, Abe T, Suzuya H, Okamoto Y, Makimoto A (2004). Cryopreservation of mobilized blood stem cells at a higher cell concentration without the use of a programmed freezer. Ann Hematol.

[34] Pasha R, Elmoazzen H, Pineault N (2017). Development and testing of a stepwise thaw and dilute protocol for cryopreserved umbilical cord blood units. Transfusion.

[35] Rubinstein P, Dobrila L, Rosenfield RE, Adamson JW, Migliaccio G, Migliaccio AR, Taylor PE (1995). Processing and cryopreservation of placental/umbilical cord blood for unrelated bone marrow reconstitution. Proc Natl Acad Sci U S A.

[36] Mrowiec ZR, Fernandez-DeLeon M, Marchioni M, Cieszynska B (2004). Comparison of controlled vs non-controlled rate freezing of umbilical cord blood units. Blood.

[37] Page KM, Zhang L, Mendizabal A, Wease S, Carter S, Gentry T, Balber AE (2011). Total colony-forming units are a strong, independent predictor of neutrophil and platelet engraftment after unrelated umbilical cord blood transplantation: a single-center analysis of 435 cord blood transplants. Biol Blood Marrow Transplant.

[38] Whaley D, Damyar K, Witek RP, Mendoza A, Alexander M, Lakey JR (2021). Cryopreservation: an overview of principles and cell-specific considerations. Cell Transplant.

[39] Jahan S, Kaushal R, Pasha R, Pineault N (2021). Current and future perspectives for the cryopreservation of cord blood stem cells. Transfus Med Rev.

